# Underwater image restoration based on dual information modulation network

**DOI:** 10.1038/s41598-024-55990-x

**Published:** 2024-03-05

**Authors:** Li Wang, Xing Li, Ke Li, Yang Mu, Min Zhang, Zhaoxin Yue

**Affiliations:** 1 School of Computer and Software, Nanjing Vocational University of Industry Technology, Nanjing, 210023 China; 2 College of information Science and Technology and College of Artificial Intelligence, Nanjing Forestry University, Nanjing, 210037 China; 3 School of Mechanical and Electrical Engineering, Nanchang Institute of Technology, Nanchang, 330000 China; 4 Department of Information Engineering, Gannan University of Science and Technology, Ganzhou, 341000 China

**Keywords:** Marine biology, Electrical and electronic engineering

## Abstract

The presence of light absorption and scattering in underwater conditions results in underwater images with missing details, low contrast, and color bias. The current deep learning-based methods bring unlimited potential for underwater image restoration (UIR) tasks. These methods, however, do not adequately take into account the inconsistency of the attenuation of different color channels and spatial regions when performing image restoration. To solve these gaps, we propose a dual information modulation network (DIMN) for accurate UIR tasks. To be specific, we design a multi-information enhancement module (MIEM), empowered by spatial-aware attention block (SAAB) and multi-scale structural Transformer block (MSTB), to guide the inductive bias of image degradation processes under nonhomogeneous media distributions. SAAB focuses on different spatial locations, capturing more spatial-aware cues to correct color deviations and recover details. MSTB utilizes the difference and complementarity between features at different scales to effectively complement the network’s structural and global perceptual capabilities, enhancing image sharpness and contrast further. Experimental results reveal that the proposed DIMN exceeds most state-of-the-art UIR methods. Our code and results are available at: https://github.com/wwaannggllii/DIMN.

## Introduction

Nowadays, the ocean is in the spotlight for its rich natural resources and great potential for applications. Due to the light interference in the underwater scene, the acquired underwater images are plagued with blurred details, distorted colors, and low contrast. Such adverse effects pose greater challenges for underwater vision tasks. As a result, underwater image restoration (UIR) technology has been implemented to boost the quality and clarity of underwater images. In our study, UIR encompasses two key aspects: super-resolution (SR) reconstruction and enhancement.

Image SR technology is designed to restore a high-resolution (HR) image from its corresponding low-resolution (LR) counterpart, thereby enlarging the region of interest for better visual effects. In recent years, leveraging the powerful representational ability of the convolutional neural network (CNN), researchers have proposed numerous image SR methods for real-world scenarios, achieving significantly improved performance^1–6^. Dong et al.^7^ pioneered a three-layer CNN for image SR, called SRCNN, outperforming traditional methods. Enlightened by this idea, plenty of tricks have emerged to further improve the network reconstruction accuracy, such as increasing the depth of the network^5,8^, widening the width of the network^9,10^, and introducing an attention mechanism^6,11^. Nevertheless, unlike natural scene images, the degradation of underwater images is more severe. To this end, researchers have also implemented some approaches for underwater SR tasks. Islam et al.^12^ constructed deep residual network-based generative models, namely SRDRM and SRDRM-GAN, for underwater SR, which can enhance underwater image resolution efficiently. Chen et al.^13^ proposed progressive attentional learning (PAL), which employs CNN with channel-wise attention and progressive learning to jointly learn a mapping from LR image to HR image. Zhang et al.^14^ introduced a new attention-guided multi-path cross-convolution neural network (AMPCNet) that enhances the model’s learning and representation of abstract information, obtaining good SR performance. Similarly, Islam et al.^15^ constructed a deep simultaneous enhancement and SR, dubbed Deep SESR, which employs two-stage residual-in-residual learning to recover image qualities.

Image enhancement technology strives to acquire clear images from degraded images for improving visual quality. Currently, underwater enhancement tasks are driven by large-scale data and have gained extensive research^16–19^. For example, Fabbri et al.^17^ adopted generative adversarial network (GAN) to improve visual quality in underwater scenes, termed underwater generative adversarial network (UGAN). Yang et al.^20^ presented a lightweight adaptive feature fusion network (LAFFNet) for underwater scenes with limited computational resources. Zhang et al.^19^ proposed a weighted wavelet visual perception fusion that corrects the color distortion of an underwater image. Huo et al.^21^ used wavelet boosting learning strategy to gradually refine underwater images in both spatial and frequency domains. It has been observed that both the above SR and enhancement methods provide better results in improving underwater image quality. However, several issues still require further attention. On the one hand, most UIR works tend to overlook the exploration of larger spatial contexts, which is directly linked to the accuracy of color correction. On the other hand, current CNN-based methods encounter challenges in establishing long-range dependencies on image features, resulting in less-than-optimal image restoration accuracy.

To alleviate the above issues, we present a new method named dual information modulation network (DIMN) for the UIR task. DIMN leverages a multi-information enhancement module (MIEM) as a backbone to progressively extrapolate information from coarse-grained to fine-grained space. In MIEM, spatial-aware attention block (SAAB) can effectively model diverse spatial location relationships, thereby enlarging spatial regions to ameliorate color cast and preserve fine details. While multi-scale structural Transformer block (MSTB) explores multi-scale structure attention mechanism to enhance the image sharpness further. Experimental results reveal that our DIMN performs competitively with state-of-the-art (SOTA) algorithms for both underwater image SR and enhancement. In brief, this study offers the following contributions:

- We propose a DIMN for accurate UIR tasks, where chained stacking MIEM can better consider the consistency of the attenuation of different color channels and spatial regions. Thanks to MIEM empowered by SAAB and MSTB, our DIMN achieves high-quality image restoration results.
- We design SAAB that explores different spatial location relationships to expand spatial-aware cues, helping to correct color deviation and enhance image details.
- We develop MSTB to generate more insightful semantic cues using a multi-scale structure attention strategy, thereby generating visually pleasing underwater results with fewer distortions and artifacts.

## Related work

### Deep learning-based UIR

Typically, deep learning-based UIR tasks can be broadly categorized into two groups: CNN and GAN. Islam et al.^12^ implemented a novel residual-in-residual CNN for underwater SR, where SRDRM-GAN incorporates a Markovian PatchGAN^22^ as their discriminator. Cherian et al.^23^ constructed a GAN-based model, called AlphaSRGAN, which is based on an alpha generative adversarial network for adversarial training of underwater image pairs. In PAL^13^, the residual attention upsampling block consisted of different convolutions to deepen the network and make the training process easier. In a paper by Wang^24^, different distillation modules were designed to aggregate local distilled information from various stages so as to attain more robust feature representations. For the underwater enhancement task, Wang et al.^25^ developed a deep CNN approach for underwater enhancement, learning strong feature representation to simultaneously achieve color rectification and haze removal. Li et al.^26^ trained the UIEB dataset using a CNN model called Water-Net for underwater image enhancement. FUnIE-GAN^27^ was a fully convolutional conditional GAN-based model for underwater image enhancement, which can enhance perceptual image quality. LAFFNet^20^ was an encoder–decoder architecture with multiple adaptive feature fusion modules, which can generate multi-scale features to recover rich image details. Apart from solving the underwater SR and enhancement tasks separately as described above, some researchers are committed to designing a unified model that can handle the UIR task in a more versatile and efficient manner. Deep SESR^15^ leveraged residual dense blocks as the backbone to facilitate improved hierarchical feature learning, obtaining good performance on underwater SR and enhancement. Sharma et al.^28^ proposed a multi-stage deep CNN for UIR, called Deep WaveNet, and proved its robustness in different tasks. Despite the promising outcomes obtained by CNN and GAN-based methods in UIR tasks, a common limitation of these methods is that they mainly emphasize on exploring local information, which may not be conducive to generating clearer images.

### Transformer-based UIR

In recent work, Transformer^29^ has gained increasing attention in UIR tasks, in particular the advantages of self-attention mechanisms in capturing long-distance dependencies and global features. Peng et al.^30^ employed U-shape Transformer network that effectively removes color artifacts and casts. Analogously, Shen et al.^31^ implemented a novel dual attention Transformer-based approach in accordance with the properties of underwater image degradation. Huang et al.^32^ designed new adaptive group attention and embedded it in Swin Transformer^?^ to focus on the dependencies between channels, showing outstanding effects in terms of color, brightness, and sharpness. Ren et al.^33^ constructed U-Net-based reinforced Swin-Convs Transformer dealing with underwater enhancement and SR, named URSCT. URSCT fused convolution to Swin Transformer to compensate for more local attention. Wang et al.^34^ constructed a novel underwater co-enhancement approach which is realized through physically guided Transformer interaction to excavate the rich semantic information. Inspired by Deep WaveNet, Wang et al.^35^ departed from the conventional CNN-based networks and instead adopted the Vision Transformer as a robust baseline for UIR, and proposed a new Transformer-based block termed URTB to solve the color degradation problem, particularly across different channels. Based on the description above, applying Transformer to the UIR task can well solve the problem of the CNN-based method’s lack of global information, while obtaining a significant improvement in recovery accuracy.

## Methods

### Network framework

**Figure 1:**
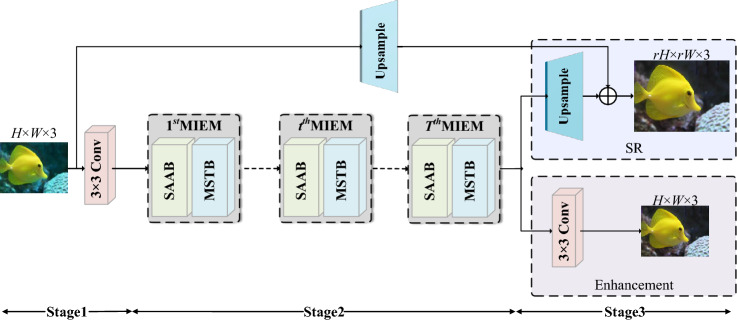
Dataflow of the proposed DIMN for accurate UIR, which consists of three stages. MIEM is enabled by SAAB and MSTB to jointly deal with attenuation inconsistencies in color channels and focus on richer spatial regions.

Figure 1 demonstrates that our DIMN consists of three stages. Stage 1 focuses on obtaining coarse-grained features, Stage 2 delves into more complex features, and Stage 3 is dedicated to restoring distorted images. Let $X \in {\mathbb {R}}^{H \times W \times \textrm{C}}$ be the distorted image, whereas $S \in {{\mathbb {R}}^{rH \times rW \times 3}}$ and $E \in {{\mathbb {R}}^{H \times W \times 3}}$ respectively belong to HR image and enhanced image. *H* and *W* represent the height and width of the image. *r* represent the scale factor, meaning that each pixel of an HR image is equivalent to the spatial extent of ${r^2}$ pixels in an LR image.

**Stage 1.** We obtain coarse-grained features from degraded underwater images using a $3\times 3$ convolution, while expanding the number of channels:

1 $$\begin{aligned} {F_0} = {H_{SFE}}\left( X \right) \end{aligned}$$

where ${H_{SFE}}\left( \cdot \right)$ is $3\times 3$ convolution operation. ${F_0} \in {{\mathbb {R}}^{H \times W \times C}}$ represent the extracted coarse-grained features, in which *C* is the number of channels.

**Stage 2.** Stage 2 is composed of *T* MIEMs, which extrapolate features from coarse-grained to fine-grained space for high-quality image restoration.

2 $$\begin{aligned} {F_t} = H_{MIEM}^t\left( {{F_{t - 1}}} \right) = H_{MIEM}^t\left( {H_{MIEM}^{t - 1}\left( { \cdots H_{MIEM}^1\left( {{F_0}} \right) \cdots } \right) } \right) \end{aligned}$$

where ${H_{MIEM}}\left( \cdot \right)$ denotes the operation of MIEM whose details are described in Section “Multi-information enhancement module (MIEM)”. ${F_t}$ is extracted fine-grained features.

**Stage 3.** In the SR tasks, an upsampling operation that is necessary to scale fine-grained features to the desired HR size. In the enhancement task, a simple $3\times 3$ convolution is utilized to produce the final enhanced image. We define the process of Stage 3 as follows:

3 $$\begin{aligned} DIMN\left( X \right) = \;\;\left\{ {\begin{array}{l} {S = {H_{UP0}}\left( {{F_t}} \right) + {H_{UP1}}\left( X \right) }\\ {E = {H_{EN}}\left( {{F_t}} \right) } \end{array}} \right. \end{aligned}$$

where $DIMN\left( \cdot \right) \in \left\{ {S,E} \right\}$ indicates the output of recovered images. ${H_{UP}}\left( \cdot \right)$ denotes the upsample operation, including a convolutional layer ($3\times 3$ convolution for ${H_{UP0}}\left( \cdot \right)$ and $5\times 5$ convolution for ${H_{UP1}}\left( \cdot \right)$ ) and a sub-pixel convolutional layer. ${H_{EN}}\left( \cdot \right)$ represents the enhancement operation, which is performed using a $3\times 3$ convolution.

We adopt ${L_1}$ loss to minimize the L-norm distance between the restored image $DIMN\left( X \right)$ and the ground truth *Y*. Given a training dataset $\left\{ {{X_m},{Y_m}} \right\} _{m = 1}^N$, here ${X_m}$ denotes the *m*-th input image and ${Y_m}$ denotes the corresponding ground-truth image. ${L_1}$ loss can be expressed as follows:

4 $$\begin{aligned} {{\mathscr {L}}}\left( \Theta \right) = \frac{1}{N}\sum _{m = 1}^N {\left\| {{Y_m} - DIMN\left( {{X_m}} \right) } \right\| _1} \end{aligned}$$

where $\Theta$ means the learnable parameters in DIMN.

### Multi-information Enhancement Module (MIEM)

As presented in Fig. 1, our proposed MIEM is driven by SAAB and MSTB, which respectively modulate spatial and global information to instruct the induction bias of the image degradation process under inhomogeneous media distribution. SAAB first extracts spatial perception information by modulating spatial location relationships. Then MSTB receives spatial information to further complement the structural and global perception of the network. SAAB and MSTB are specifically depicted in Sections “Spatial-aware attention block (SAAB)” and “Multi-scale structural transformer block (MSTB)”, respectively.

### Spatial-aware attention block (SAAB)

**Figure 2:**
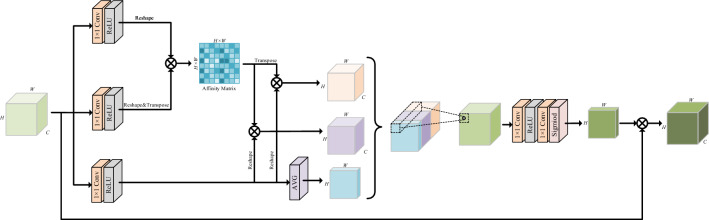
Diagram of our proposed SAAB, modeling spatial location relationships of the images to abstract richer content features.

The scattering of underwater light and particles in the water causes more severe distortion in distant scenes and less distortion in nearby scenes^31^. Therefore, for non-uniformly degraded underwater imagery, it is critical to model the spatial location relationships of the images that extend the restoration of content-rich features. As displayed in Fig. 2, we devise a SAAB that explores spatial-aware information to improve the attention of spatial regions, resulting in better enhancement of target features and reduction of visual artifacts.

Given the input features ${F_{t - 1}} \in {{\mathbb {R}}^{H \times W \times C}}$ of the *t*-th MIEM, its output spatial-aware features $F_t^{SAAB} \in {{\mathbb {R}}^{H \times W \times C}}$ after SAAB. To be specific, ${F_{t - 1}} \in {{\mathbb {R}}^{H \times W \times C}}$ is initially delivered into $1\times 1$ convolution and reshaped to acquire feature maps $P_q^t \in {{\mathbb {R}}^{\left( {H \times W} \right) \times C}}$ and ${P_k} \in {{\mathbb {R}}^{C \times \left( {H \times W} \right) }}$. Then we compute spatial affinity $r_{_{i,j}}^t$ from *i*-th to *j*-th through matrix multiplication:

5 $$\begin{aligned} r_{_{i,j}}^t= P_{q,i}^t \cdot P_{k,j}^t \end{aligned}$$

where $P_{q,i}^t$ and $P_{k,j}^t$ are the *i*-th and *j*-th deep pixel of local features. Similarly, we can get spatial affinity from *j*-th to *i*-th as $r_{_{j,i}}^t$. Therefore, we can get an affinity matrix ${R^t} \in {{\mathbb {R}}^{\left( {H \times W} \right) \times \left( {H \times W} \right) }}$ among all positions.

For the purpose of learning the attention of the *i*-th feature position, we also include the feature itself to utilize the global information and local original information relative to that feature. On the one hand, ${F_{t - 1}} \in {{\mathbb {R}}^{H \times W \times C}}$ is first passed through another convolutional layer to obtain a feature map $P_v^t \in {{\mathbb {R}}^{H \times W \times C}}$ and reshape it to $P_v^t \in {{\mathbb {R}}^{\left( {H \times W} \right) \times C}}$. Then we can attain spatial relation-aware features $P_Q^t \in {{\mathbb {R}}^{H \times W \times C}}$ through matrix multiplication:

6 $$\begin{aligned} P_{Q,i}^t = P_{v,i}^t \cdot r_{i,j}^t \end{aligned}$$

Analogously, the spatial relation-aware features $P_K^t \in {{\mathbb {R}}^{H \times W \times C}}$ at the *j*-th feature position can be expressed as $P_{K,j}^t = P_{v,j}^t \cdot r_{j,i}^t$. On the other hand, we leverage the global average pooling operation to suppress the feature map $P_v^t \in {{\mathbb {R}}^{H \times W \times C}}$ along the channel dimension to 1 and obtain spatial features $P_V^t \in {{\mathbb {R}}^{H \times W \times 1}}$. Considering these three types of information fall outside the same feature domain, we concatenate them and embed them to get spatial-aware modulation coefficients ${\varpi ^t} \in {{\mathbb {R}}^{H \times W \times 1}}$:

7 $$\begin{aligned} {\varpi ^t} = \Phi \left( {\left[ {P_Q^t,P_K^t,P_V^t} \right] } \right) \end{aligned}$$

where $\Phi \left( \cdot \right)$ indicates the embedding function, implemented by two $1\times 1$ convolutions, ReLU activation, and a Sigmoid function. Finally, we merge ${F_{t - 1}}$ and ${\varpi ^t}$ to output the final spatial-aware features $F_{SAAB}^t \in {{\mathbb {R}}^{H \times W \times C}}$:

8 $$\begin{aligned} F_{SAAB}^t = {\varpi ^t} \cdot {F_{t - 1}}= \sum _{i = 1}^{H \times W} \varpi _{i,j}^t{F_{t - 1,j}} \end{aligned}$$

In this way, we can mine the non-local context to refine the features at each spatial position for inferring attention through a learnable model. The implementation of SAAB is depicted in Algorithm 1.

**Algorithm 1:**
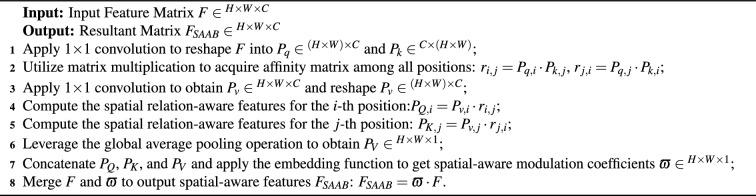
The implementation of spatial-aware attention block.

### Multi-scale structural transformer block (MSTB)

**Figure 3:**
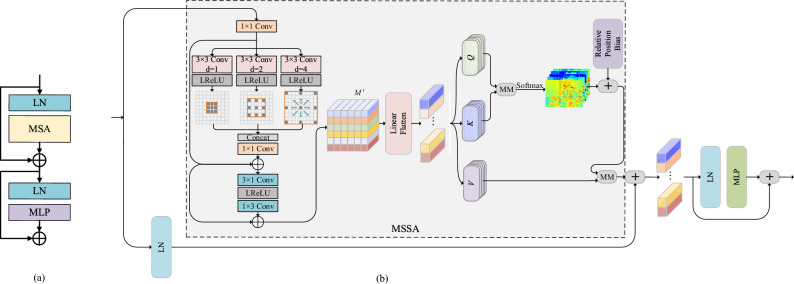
(**a**) Standard Transformer; (**b**) Our proposed MSTB, which is equipped with MSSA to capture deeper semantic clues.

Despite the prolific literature on Transfomer-based UIR methods, existing works suffer from severe color distortions and missing details. It is known that different dilated convolutions can provide different receptive field sizes, thereby extending global coherence to alleviate visual artifacts caused by color bias. As depicted in Fig. 3a, a standard Transformer with multi-head self-attention (MSA) mechanism can efficiently model long-range dependence features to improve image sharpness. Enlightened by these works, we propose a MSTB that strengthens the focus on more severely attenuated spatial and color channels, improving recovery accuracy. As depicted in Fig. 3b, we commence by encoding multi-scale features through the utilization of different dilation convolutions. Following this, we apply asymmetric convolutions to extract structural information both horizontally and vertically. Ultimately, these processed features are passed into the Transformer, enabling the capture of more profound semantic cues for enhanced image restoration.

Knowing that the input to the MSTB is $F_{SAAB}^t$, we define the multi-scale and structural feature extraction part as:

9 $$\begin{aligned} {M^t} = H_{MS}^t\left( \left[ f_{3 \times 3}^{d = 1}\left( {F_{SAAB}^t} \right) ,f_{3 \times 3}^{d = 2}\left( {F_{SAAB}^t} \right) ,f_{3 \times 3}^{d = 4}\left( {F_{SAAB}^t} \right) \right] \right) \end{aligned}$$

where ${M^t} \in {{\mathbb {R}}^{H \times W \times C}}$ indicates the extracted diversity features. ${H_{MS}}\left( \cdot \right)$ indicates the embedding function, implemented by 1$\times$1 convolution, $3\times 1$convolution, and $1\times 3$ convolution followed by LReLU activation. ${f_{3 \times 3}}\left( \cdot \right)$ denotes $3\times 3$ convolution, where the superscript is the dilation rate. As shown in Fig. 3b, we unfold the diverse features ${M^t}$ and compute query *Q*, key *K*, and value *V* using a fully connected layer, which can be expressed by:

10 $$\begin{aligned} {Q} = {W_q}{M^t},\quad K = {W_k}{M^t},\quad V = {W_v}{M^t} \end{aligned}$$

The attention matrix *Att*(*Q*, *K*, *V*) is computed as:

11 $$\begin{aligned} {{Att(Q,K,V)}}=\hbox{softmax}\left( {\frac{{{M^t}{W_q}W_k^T{M^t}^T}}{{\sqrt{{d_k}}}}} \right) {M^t}{W_v} = \hbox{softmax}\left( {\frac{{Q{K^T}}}{{\sqrt{{d_k}} }}} \right) \mathrm{{V}} \end{aligned}$$

where ${W_q}$, ${W_k}$, and ${W_v}$ indicate the weight matrices of queries, keys, and values. $\sqrt{{d_k}}$ denotes normalization factor. We refer to the above operation as multi-scale structure attention (MSSA), and its pseudo-code is reported in Algorithm 2. The output of MSTB is defined as:

12 $$\begin{aligned} \begin{array}{l} {{{\bar{F}}}^t} = MSSA\left( {F_{SAAB}^t} \right) + LN\left( {F_{SAAB}^t} \right) \\ {F^t} = MLP\left( {LN\left( {{{{\bar{F}}}^t}} \right) } \right) + {{{\bar{F}}}^t} \end{array} \end{aligned}$$

where MLP denotes multi-layer perceptron and LN is layer normalization operation.

**Algorithm 2:**
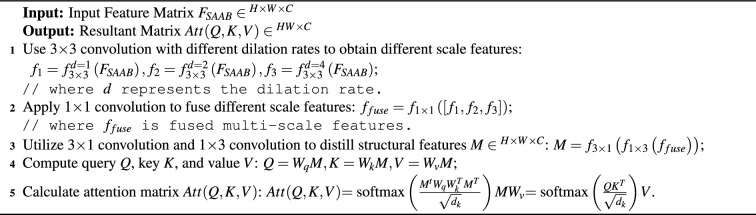
The implementation of multi-scale structure attention.

## Experiments

### Datasets and metrics

We adopt publicly available UIR datasets to train our proposed DIMN, including USR-248^12^, UFO-120^15^, EUVP^27^, and UIEB^26^. Specifically, we respectively use USR-248 and UFO-120 datasets to conduct underwater SR task. The USR-248 dataset comprises 1060 pairs for training and 248 pairs for testing. Among them, LR images are acquired by downsampling HR images using scale factors of $\times$2, $\times$4, and $\times$8 through bicubic interpolation, with the addition of 20% Gaussian noise. The UFO-120 dataset comprises 1500 pairs for training and 120 pairs for testing with scale factors of $\times$2, $\times$3, and $\times$4. In the underwater enhancement task, the EUVP dataset comprises 11,435 paired images for training and 515 paired images for testing. The UIEB dataset comprises 890 pairs of images, with 800 pairs allocated for training and 90 pairs for testing. We employ reference metrics (mean-squared error(MSE), peak signal-to-noise ratio(PSNR), structure similarity index(SSIM), underwater image quality measure(UIQM)^36^, natural image quality evaluator(NIQE)^37^) and non-reference metrics (patch-based contrast quality index(PCQI)^38^, underwater image sharpness measure(UISM)^36^, average entropy(E), and underwater color image quality evaluation(UCIQE)^39^) to assess experimental results. Particularly, UIQM includes three underwater image attribute measures: underwater image colorfulness measure (UICM), UISM, and underwater image contrast measure (UIConM), which provide a comprehensive assessment of restoration images. UIQM can be calculated as follows:

13 $$\begin{aligned} UIQM = {c_1} \times UICM + {c_2} \times UISM + {c_3} \times UIConM \end{aligned}$$

where ${c_1}$, ${c_2}$, and ${c_3}$ denote scale parameters that are set to 0.0282, 0.2953, and 3.5753.

We utilize the Adam optimizer to minimize the objective function, with optimizer parameters set as ${\beta _1} = 0.9$, ${\beta _2} =0.999$, and $\varepsilon = {10^{ - 8}}$. The initial learning rate is fixed at $1\hbox{e}{-}3$ and is halved every 100 epochs. To accommodate memory constraints, each batch comprises 32 LR patches of size $50\times 50$ for the SR task and 16 patches of size $100\times 100$ for the enhancement task. The implementation of our model utilizes the PyTorch framework and is executed on NVIDIA TESLA V100 GPU.

### Ablation study

In this section, two full-reference image quality assessment indexes (PSNR and SSIM) and four reference-free image quality assessment indexes (UIQM, NIQE, MA^40^, and PI^41^) are employed to quantitatively compare the restoration results of different models. To explicitly demonstrate how our proposed components enhance the restoration results, four experiments of the relevant components are performed. We first get rid of SAAB and MSTB in turn, which are respectively called DIMN w/o SAAB and DIMN w/o MSTB. Then, we remove the MSSA in MSTB, making MSTB a standard Transformer (Fig. 3a) and naming it DIMN w/o M. Finally, we substitute $3\times 3$ and $5\times 5$ convolutions for the multi-scale part and designated it DIMN w P. Here, FLOPs is computed at a $640\times 480$ HR image.

#### Impact of SAAB and MSTB

**Figure 4:**
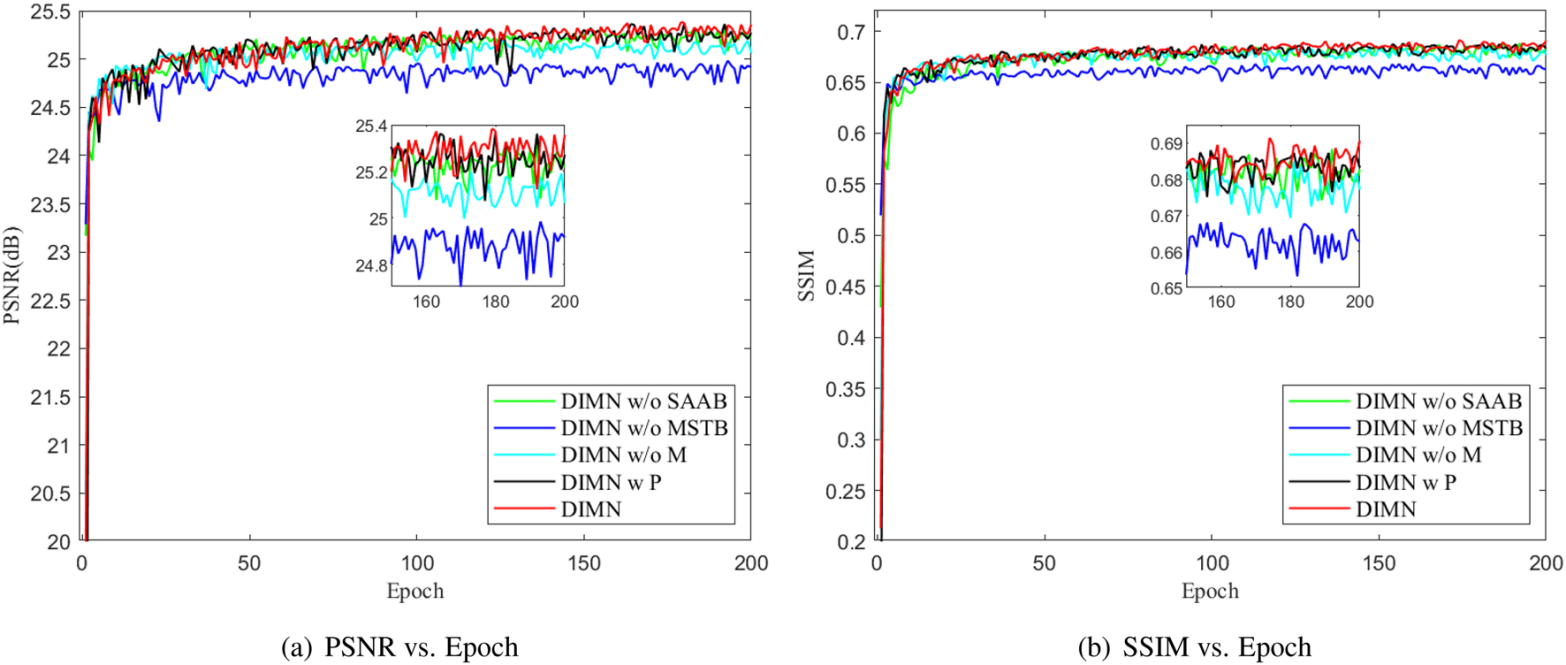
Convergence results for different models on UFO-120 dataset with scale factor $\times$4.

The experimental results are reported in Table 1. One can see that the model enabled by SAAB and MSTB attains favorable performance, which improves 0.05 dB and 0.0043 over DIMN w/o SAAB, as well as 0.37 dB and 0.0139 over DIMN w/o MSTB. On the one hand, the absence of SAAB makes it difficult to provide sufficient spatial information, which is detrimental to producing high-quality and high-resolution images. On the other hand, the introduction of MSTB can provide large gains in expanding the receptive field, preserving structural information, and modeling global features, thus effectively addressing the negative effects of color bias and distortion. Also, Fig. 4 depicts the convergence results for different components, we can observe that the aggregation of SAAB and MSTB contributes to stable network convergence.

**Table 1:** Ablation studies of proposed components on UFO-120 dataset with scale factor $\times$4.

Methods	Params	FLOPs	PNSR	SSIM	UIQM	NIQE$\nabla$	MA	PI$\nabla$
DIMN w/o SAAB	841K	16.1G	25.30	0.7037	2.9533	66.454	3.8503	6.3976
DIMN w/o MSTB	135K	2.60G	24.98	0.6941	2.8243	65.611	3.7698	6.3956
DIMN w/o M	352K	6.70G	25.17	0.7023	2.8691	66.116	3.7188	6.4464
DIMN w P	942K	18.1G	25.34	0.7032	2.9362	66.971	**3**.**8814**	6.4079
DIMN	942K	18.1G	**25**.**35**	**0**.**7080**	**2**.**9587**	**64**.**013**	3.8571	**6**.**2721**

Significant values are in bold.

#### Impact of components in MSTB

**Figure 5:**
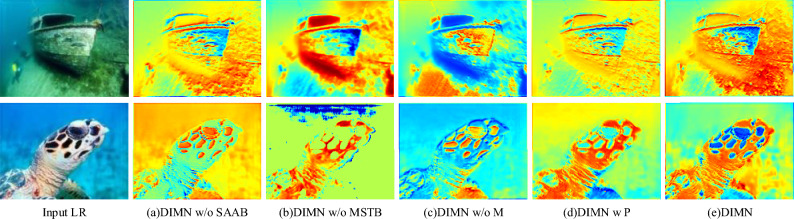
Visualization results of average feature maps on different methods.

Compared to DIMN, DIMN w/o M is severely degraded in all metrics, with NIQE decreasing from 64.013 to 66.116, MA decreasing from 3.8571 to 3.7188, and PI decreasing from 62.721 to 64.464. This is because the multi-scale and structural feature extraction part can leverage different scale and structural cues to modulate more delicate features for subsequent Transformer operations. When compared to DIMN w/P, DIMN holds a notably more substantial advantage. Although DIMN is slightly behind in MA score, the difference is only 0.0243. Figure 5 illustrates the feature heatmap of different methods, visualizing how they retain the detailed features of the underwater image. In the feature heatmap, the red color indicates that the network is paying more attention to the target area. Without the support of the multi-scale and structural feature extraction component (DIMN w/o M), the attention on the discriminant region is also significantly reduced. Compared to DIMN, DIMN w P can focus on the target area better, but at a lower intensity. Importantly, DIMN supported by SAAB and MSTB responds more positively to the target object, allowing more effort to be allocated to modulating these areas, thus resulting in richer texture detail for high-quality image restoration.

#### Impact of number of MIEMs

In Table 2, we verify the selection of the number of MIEM, in which the numbers are set to $T=2$, 4, and 6, respectively. Obviously, the improvement in recovery accuracy becomes small when the value of *T* exceeds 4. Taking into account the trade-off between network complexity and restoration performance, we have opted for $T=4$ as the number of MIEMs.

**Table 2:** Results for different numbers of MIEM on USR-248 dataset with scale factor $\times$2.

T	Params	FLOPs	PNSR	SSIM	UIQM
2	464K	35.6G	29.68	0.8224	2.7400
4	919K	70.5G	**29**.**96**	**0**.**8327**	2.7829
6	1.37M	105.3G	**29**.**96**	0.8318	**2**.**7835**

Significant values are in bold.

Overall, the ablation studies reveal the effectiveness and robustness of our proposed MIEM incorporating SAAB and MSTB.

### Comparison with underwater SR methods

#### Evaluation on USR-248 dataset

We compare the proposed DIMN with some SOTA methods on the USR-248 dataset, including SRCNN^7^, VDSR^5^, EDSRGAN^42^, SRGAN^43^, SRResNet^43^, ESRGAN^44^, SRDRM^12^, SRDRM-GAN^12^, PAL^13^, and AMPCNet^14^. As Table 3 reports, our DIMN exhibits competitive advantages across all image quality indexes with lower model complexity. Compared to AMPCNet, our DIMN has demonstrated a distinct improvement of 0.27 dB, 0.36 dB, and 0.16 dB on PSNR. More importantly, the superiority of our proposed method becomes more obvious as the scale factor increases.

**Table 3:** Quantitative comparison with the best-published methods on USR-248 dataset.

Scale	Method	FLOPs (G)	Params (M)	PSNR (dB)	SSIM	UIQM
$\times$2	SRCNN^7^	21.30	0.06	26.81	0.76	2.74
	VDSR^5^	205.28	0.67	28.98	0.79	2.57
	EDSRGAN^42^	273.34	1.38	27.12	0.77	2.67
	SRGAN^43^	377.76	5.95	28.05	0.78	2.74
	SRResNet^43^	222.37	1.59	25.98	0.72	–
	ESRGAN^44^	4274.68	16.70	26.66	0.75	2.70
	SRDRM^12^	203.91	0.83	28.36	0.80	**2**.**78**
	SRDRM-GAN^12^	289.38	11.31	28.55	**0**.**81**	2.77
	PAL^13^	203.82	0.83	28.41	0.80	–
	AMPCNet^14^	–	1.15	29.54	0.80	2.77
	DIMN (Ours)	70.50	0.92	**29**.**81**	**0**.**81**	2.71
$\times$4	SRCNN^7^	21.30	0.06	23.38	0.67	2.38
	VDSR^5^	205.28	0.67	25.70	0.68	2.44
	EDSRGAN^42^	206.42	1.97	21.65	0.65	2.40
	SRGAN^43^	529.86	5.95	24.76	0.69	2.42
	SRResNet^43^	85.49	1.59	24.15	0.66	–
	ESRGAN^44^	1504.09	16.70	23.79	0.66	2.38
	SRDRM^12^	291.73	1.90	24.64	0.68	2.46
	SRDRM-GAN^12^	377.20	12.38	24.62	0.69	2.48
	PAL^13^	303.42	1.92	24.89	0.69	–
	AMPCNet^14^	–	1.17	25.90	0.66	**2**.**58**
	DIMN (Ours)	18.07	0.94	**26**.**26**	**0**.**70**	2.50
$\times$8	SRCNN^7^	21.30	0.06	19.97	0.57	2.01
	VDSR^5^	205.28	0.67	23.58	0.63	2.17
	EDSRGAN^42^	189.69	2.56	19.87	0.58	2.12
	SRGAN^43^	567.88	5.95	20.14	0.60	2.10
	SRResNet^43^	51.28	1.59	19.26	0.55	–
	ESRGAN^44^	811.44	16.70	19.75	0.58	2.05
	SRDRM^12^	313.68	2.97	21.20	0.60	2.18
	SRDRM-GAN^12^	399.15	13.45	20.25	0.61	2.17
	PAL^13^	325.51	2.99	22.51	0.63	–
	AMPCNet^14^	–	1.25	23.83	0.62	**2.25**
	DIMN (Ours)	4.97	1.03	**24**.**00**	**0**.**64**	2.18

Significant values are in bold.

**Figure 6:**
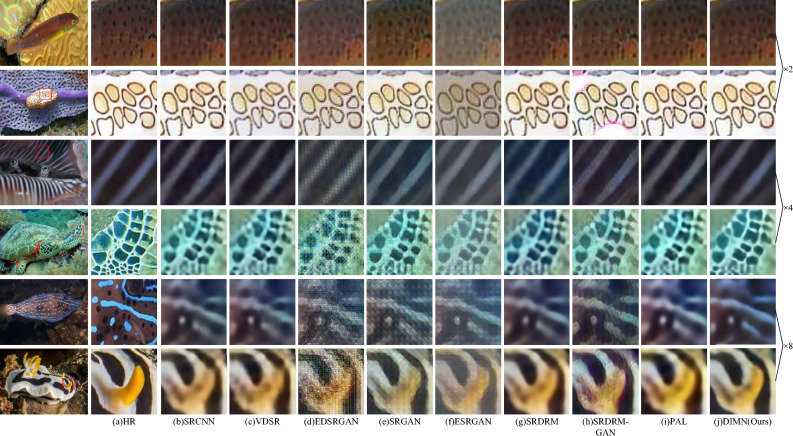
Visual comparison of our proposed DIMN against popular works on USR-248 dataset.

Figure 6 exhibits a selection of SR results on USR-248 dataset. It is evident that our method produces more favorable results, with visual effects that closely resemble HR images. Clearly, SRDRM, SRDRM-GAN, and PAL exhibit substantial blurring and distortion, while our DIMN outperforms them by recovering superior edge and texture details. This is attributed to the ability of our approach to effectively integrate both local and global information, resulting in higher resolution and sharper images.

#### Evaluation on UFO-120 dataset

**Figure 7:**
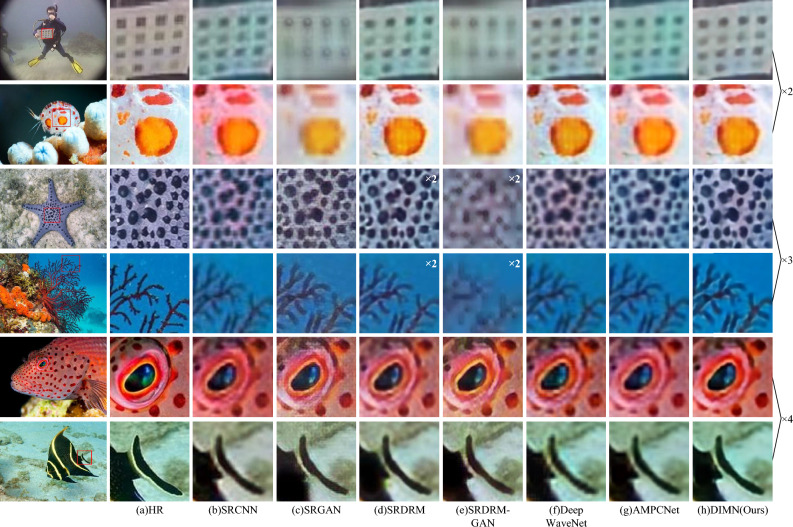
Visual comparison of our proposed DIMN against popular works on UFO-120 dataset.

We carry out a comparison of our DIMN with SRCNN^7^, SRGAN^43^, SRDRM^12^, SRDRM-GAN^12^, Deep SESR^15^, Deep WaveNet^28^, AMPCNet^14^, and URSCT^33^ using both quantitative and qualitative metrics. Quantitative and qualitative results are respectively presented in Table 4 and Fig. 7.

**Table 4:** Quantitative comparison with the best-published methods on UFO-120 dataset.

Method	PSNR (dB)			SSIM			UIQM		
	$\times$2	$\times$3	$\times$4	$\times$2	$\times$3	$\times$4	$\times$2	$\times$3	$\times$4
SRCNN^7^	24.75	22.22	19.05	0.72	0.65	0.56	2.39	2.24	2.02
SRGAN^43^	**26**.**11**	23.87	21.08	0.75	0.70	0.58	2.44	2.39	2.56
SRDRM^12^	24.62	–	23.15	0.72	–	0.67	2.59	–	2.57
SRDRM-GAN^12^	24.61	–	23.26	0.72	–	0.67	2.59	–	2.55
Deep SESR^15^	25.70	26.86	24.75	0.75	0.75	0.66	**3**.**15**	2.87	2.55
Deep WaveNet^28^	25.71	25.23	25.08	0.77	**0**.**76**	0.74	2.99	**2**.**96**	**2**.**97**
AMPCNet^14^	25.24	25.73	24.70	0.71	0.70	0.70	2.93	2.85	2.88
URSCT^33^	25.96	–	23.59	**0.80**	–	0.66	–	–	–
DIMN (Ours)	25.96	**26**.**60**	**25**.**48**	0.75	**0.76**	0.71	3.02	2.92	2.92

Significant values are in bold.

Table 4 reveals that our DIMN consistently delivers both optimal and sub-optimal performance when compared to well-established underwater SR methods. Despite a marginal underperformance in UIQM, noteworthy improvements have been observed in terms of both PSNR and SSIM. Accordingly, in the case of $\times$4, our proposed method has outperformed Deep SESR and Deep WaveNet by an improvement of 2.95% and 1.59% in PSNR. In comparison to Transformer-based methods like URSCT, our work has demonstrated a notable improvement, with an increase of 8.01% in PSNR and 7.58% in SSIM. Figure 7 further demonstrates that our method successfully rectifies color deviations, enhances detail information, and improves image contrast. SRDRM-GAN and AMPCNet fail to remove color casts and reconstruct texture detail.

### Comparison with underwater enhancement methods

#### Evaluation on EUVP dataset

**Figure 8:**
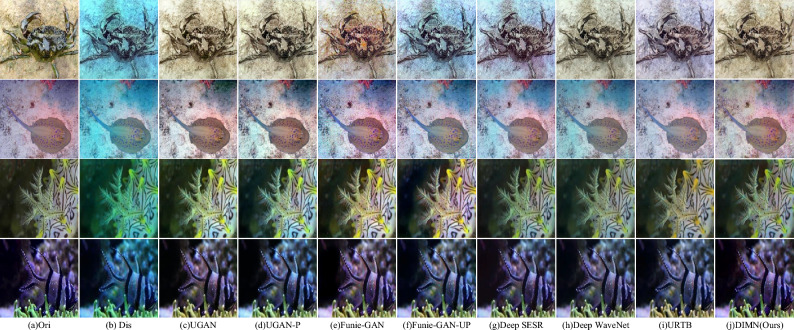
Visual comparison of the proposed DIMN against popular works on EUVP dataset.

For the underwater enhancement task, we perform a comparison of our DIMN against some of the best-published methods on the EUVP dataset. The corresponding quantitative outcomes are presented in Table 5, while the qualitative results are depicted in Fig. 8, respectively. As can be seen from Table 5, our proposed method demonstrates superior performance across the majority of quantitative metrics. For instance, our DIMN achieves improvements of no less than 5.96% and 2.38% in PSNR and SSIM, respectively. Even though UIQM, NIQE, PCQI, and E lag behind URTB, they still demonstrate competitive performance. Our method mainly employs spatial-aware and multi-scale structural features to deal with detail blurring and color casts, thus better restoration accuracy can be obtained.

**Table 5:** Quantitative comparison against the best-published methods on EUVP dataset.

Methods	PSNR	SSIM	UIQM	NIQE$\nabla$	PCQI	UISM	VIF	E$\nabla$
UGAN^17^	26.55	0.80	2.89	49.90	0.700	6.84	0.402	7.52
UGAN-P^17^	26.54	0.80	2.93	50.17	0.704	6.83	0.400	7.54
Funie-GAN^27^	26.22	0.79	2.97	50.51	0.706	6.90	0.384	7.55
Funie-GAN-UP^27^	25.22	0.78	2.93	52.87	0.702	6.86	0.394	7.50
Deep SESR^15^	27.08	0.80	**3**.**09**	55.68	0.679	**7**.**06**	0.384	7.40
Deep WaveNet^28^	28.62	0.83	3.04	44.89	0.694	**7**.**06**	0.438	7.38
URTB^35^	29.02	0.84	2.98	**43**.**75**	**0**.**849**	6.57	0.651	**7**.**14**
DIMN (Ours)	**30**.**75**	**0**.**86**	2.74	48.59	0.835	**7**.**06**	**0**.**750**	7.45

$\nabla$ denotes lower is better. Significant values are in bold.

**Figure 9:**
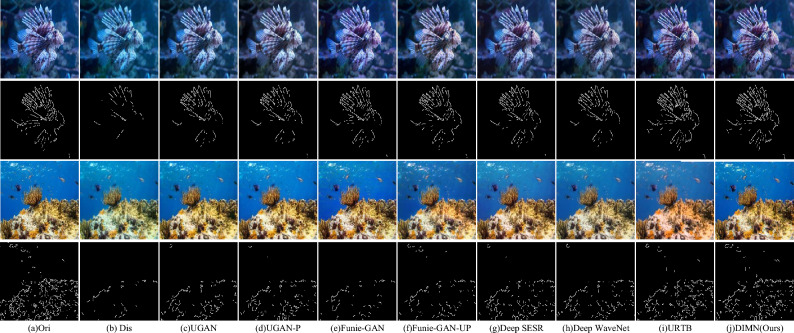
Canny edge detection on EUVP dataset.

In Fig. 8, we can notice that Funie-GAN and URTB result in over-saturation, while Funie-GAN-UP and Deep SESR struggle to remove color casts. Contrastingly, the proposed DIMN excels in color restoration and contrast enhancement. This can be attributed to the network’s robust local and global learning capabilities, allowing it to address the variation in attenuation in different color channels and spatial areas. Additionally, we have incorporated the Canny^45^ algorithm in Fig. 9 to evaluate the extent of improvement in image clarity. Figure 9 depicts the enhanced results and their corresponding edge maps. We can see that the edges of distorted images are difficult to detect due to strong scattering. Observing from Fig. 9j, the proposed DIMN reproduces more edge detection features and contains almost all contours. This reveals that the proposed method can effectively eliminate color artifacts and recover more structural information, which is advantageous for producing visually satisfying results.

#### Evaluation on UIEB dataset

**Figure 10:**
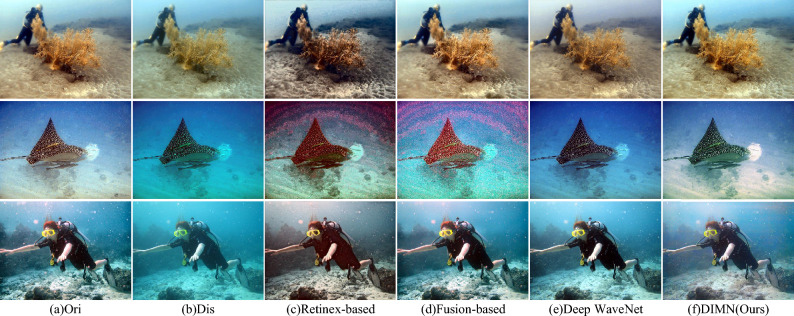
Visual comparison of the proposed DIMN against popular works on UIEB dataset.

For a fair comparison, we train and test the UIEB following the setup described in^28^. Quantitative and qualitative results are listed in Table 6 and Fig. 10, respectively. According to Table 6, it is evident that our proposed scheme harvests the best performance. Particularly in the PSNR metric, compared with prevailing UIR methods such as Deep SESR, Deep WaveNet, and URTB, our DIMN demonstrates significant improvements of 31%, 1.15% and 0.51%, respectively.

**Table 6:** Quantitative comparison against the best-published methods on UIEB dataset.

Methods	MSE	PSNR	SSIM
Fusion-based^46^	0.91	21.23	0.78
Retinex-based^47^	1.34	17.66	0.61
GDCP^48^	3.33	13.86	0.55
Water CycleGAN^49^	1.72	15.75	0.52
DenseGAN^50^	1.21	17.28	0.44
WaterNet^26^	0.79	19.11	0.79
Deep SESR^15^	1.70	16.65	0.57
Deep WaveNet^28^	0.60	21.57	0.80
URTB^35^	–	21.71	0.83
DIMN (Ours)	**0**.**56**	**21**.**82**	**0**.**84**

Significant values are in bold.

In Fig. 10, one can see that the Retinex-based method exhibits color oversaturation in the enhanced images. Fusion-based and Deep WaveNet fail to remove color casts and have poor visual effects. In contrast, the results enhanced by our method are more faithful to the original image, benefitting from the joint learning of spatial location and global cues by SAAB and MSTB.

## Conclusion

In this study, we present an accurate and efficient DIMN, empowered by a sequence of MIEMs, for the UIR task. MIEM serves as the backbone of the network that effectively handles attenuation inconsistencies across color channels and spatial regions, thereby removing color artifacts, enhancing contrast, and restoring detail. In MIEM, SAAB can model different spatial location relationships to explore content-rich features, while MSTB utilizing multi-scale structure attention scheme strengthens the focus on more severely attenuated spatial and color channels to boost recovery accuracy further. Experimental results reveal that the competitiveness of our DIMN when compared to SOTA approaches, and ablation studies confirm the contributions of our proposed MIEM comprising SAAB and MSTB.

## Data Availability

USR-248, UFO-120, and EUVP datasets are available from: https://irvlab.cs.umn.edu/resources. UIEB dataset is available from: https://li-chongyi.github.io/proj_benchmark.html.
